# Fractional anisotropy in children with dystonia or spasticity correlates with the selection for DBS or ITB movement disorder surgery

**DOI:** 10.1007/s00234-015-1639-9

**Published:** 2016-01-12

**Authors:** Daniel E. Lumsden, Jonathan Ashmore, Gareth Ball, Geoffrey Charles-Edwards, Richard Selway, Keyoumars Ashkan, Jean-Pierre Lin

**Affiliations:** Complex Motor Disorders Service, Evelina Children’s Hospital, Guy’s & St Thomas’ NHS Foundation Trust, Lambeth Palace Road, London, SE1 7EH UK; Imaging Sciences and Biomedical Engineering, King’s College London, London, UK; Centre for the Developing Brain, King’s College London, London, UK; Medical Physics, St Thomas’ Hospital, Guy’s and St Thomas’ NHS Foundation Trust, London, UK; Functional Neurosurgery, King’s College Hospital, King’s College Hospital NHS Foundation Trust, London, UK; Clinical Neuroscience, Institute of Psychiatry, London, UK

**Keywords:** Dystonia, Spasticity, Deep brain stimulation, Intrathecal baclofen, Fractional anisotropy

## Abstract

**Introduction:**

There is increasing interest in neurosurgical interventions for hypertonicity in children and young people (CAYP), which often presents with a mixture of dystonia and spasticity. Significant spasticity would usually be considered a contraindication for deep brain stimulation (DBS) and more suitably treated with intrathecal baclofen (ITB). We aimed to explore whether white matter microstructure, as measured by Fractional Anisotropy (FA), differed between CAYP selected for DBS compared to ITB surgery.

**Methods:**

We retrospectively analysed Diffusion Tensor Imaging for 31 CAYP selected for DBS surgery (14 primary dystonia, 17 secondary dystonia) and 10 CAYP selected for ITB surgery. A voxel-wise comparison of FA values was performed using tract-based spatial statistics, comparing primary and secondary dystonia groups to the ITB group, and the two dystonia groups.

**Results:**

Widespread areas of reduced FA were demonstrated in ITB compared to either DBS group and in CAYP with secondary compared to primary dystonia. These changes were not restricted to motor pathways. Region of interest (ROI) analysis from the corticospinal tract (CST) demonstrated groupwise differences but overlapping values at the individual level.

**Conclusions:**

DTI measures may contribute to decision making for CAYP selection for movement disorder surgery. Significant differences in CAYP with secondary dystonia selected for DBS surgery compared to CAYP selected for ITB pump implants, suggesting that more extensive white matter injury may be a feature of the spastic motor phenotype. Altered white matter microstructure could potentially explain the reduced responsiveness to interventions such as DBS in secondary compared to primary dystonia.

## Introduction

Hypertonicity in childhood has been variably described by the terms “spasticity”, “dystonia” and “rigidity”. Spasticity can be defined as “resistance to externally imposed movement increases with increasing speed of stretch and varies with the direction of joint movement,” whilst dystonia is defined as “a movement disorder in which involuntary sustained or intermittent muscle contractions cause twisting and repetitive movements, abnormal postures, or both” [1]. In childhood, spasticity and dystonia may be coincident, particularly when hypertonicity arises due to an extrinsic insult/injury to the brain, e.g. cerebral palsy (CP) or acquired brain injury [2]. Discriminating between and quantifying the extent of spasticity and/or dystonia in a child may be challenging, as can determining the relative contribution of each to the child’s functional impairments. Dystonia in cerebral palsy is also frequently accompanied by chorea and/or athetosis, collectively categorized as “dyskinetic” CP.

A number of neurosurgical interventions are available in hypertonicity management, with intrathecal baclofen (ITB) and deep brain stimulation (DBS) used most widely. DBS of the globus pallidus interna (GPi) has been widely adopted in the management of primary dystonia in adulthood, with sustained beneficial effects seen up to several years following initial implantation [3, 4]. DBS has been shown to be less beneficial in secondary dystonia [5]. Baclofen is an agonist of the inhibitory neurotransmitter gamma-aminobutyric acid (GABA) [6]. ITB has become well established in the management of spasticity [7], particularly in the setting of CP. ITB has been less studied in the management of dystonia though positive findings have been reported [8].

In primary dystonia, DBS, where available, is universally offered rather than ITB. For secondary dystonia, the choice between DBS and ITB is strongly influenced by the degree of spasticity and corticospinal impairment. Significant impairment of the corticospinal tract (CST), as indicated by abnormal Central Motor Conduction Time (CMCT) and other measures [9], would be considered a contraindication to DBS surgery and an indication for ITB pump implant surgery. Patients undergoing DBS surgery are those with a relatively pure dystonic motor phenotype, whilst those undergoing ITB surgery would represent those with a significant spastic component.

Diffusion Tensor Imaging (DTI) is an MRI technique which provides information about white matter microstructure and is derived from the differential diffusion of water molecules [10]. In white matter regions, water diffuses more easily along the orientations of fibres, with the greatest restriction at right angles to this direction. The CST has been the subject of a number of DTI studies, particularly in the setting of spastic CP [11]. Abnormal CST metrics have been described in comparison to healthy controls, with the degree of abnormality correlating with the severity of motor impairment.

Direct comparisons of DTI metrics between spastic and other subtypes of CP are currently limited. Two studies from one group, one using a region-of-interest (ROI) and one using an atlas-based approach, have compared children with spastic and dyskinetic CP [12, 13]. These studies demonstrated greater white matter disruption in the dyskinetic group, though motor impairment was more severe in the children with spasticity in the atlas-based study (with no details of motor function in the ROI-based study).

The potential application of diffusion tensor imaging-based techniques to the field of movement disorder surgery is an area of active research. This includes improving target selection, improving the understanding of the distributed network activated by DBS and also monitoring the effects of the intervention. We aimed to determine if quantitative white matter differences could be found between children and young people (CAYP) undergoing DBS and those undergoing ITB at our centre using tract-based spatial statistics (TBSS) analysis. This retrospective analysis was intended to explore the possible utility of DTI-derived metrics as part of the clinical assessment of hypertonic children proposed as candidates for movement disorder surgery and also to explore differences in white matter microstructure between the predominantly dystonic and predominantly spastic motor phenotypes.

## Materials and methods

The case notes and imaging records of all CAYP referred to our service between July 2008 and January 2012 for evaluation prior to possible neurosurgical intervention were reviewed. CAYP who had undergone suitable DWI imaging during their clinical assessment where identified for further processing. Suitable imaging sequences were available for 31 CAYP put forward for DBS surgery (14 with a diagnosis of primary dystonia, 17 with a diagnosis of secondary dystonia) and 10 CAYP put forward for ITB surgery (Table 1). Median age at time of scan was 9.5 years (range 2.75 to 17.75) and did not differ between groups (Kruskal-Wallis test, *p* < 0.05). Gross Motor Function Classification scores (GMFCS) [14] were lower in the primary DBS groups compared to secondary dystonia DBS and ITB groups (chi-squared test, *p* < 0.05) but did not differ between the secondary dystonia DBS compared to ITB group.

**Table 1:** Clinical details of 41 CAYP involved in the study

Surgical intervention	Diagnostic classification	Diagnosis	GMFCS	Age at scan
Deep brain stimulation	Primary dystonia	DYT1 −ve	2	8.75
		Dystonia myoclonus syndrome	1	16.75
		DYT1 −ve	5	16
		DYT1 −ve	1	17.25
		DYT1 −ve	2	10
		DYT1 −ve	2	13.9
		DYT1 +ve	3	11.6
		DYT11 +ve	2	9.5
		DYT1 −ve	2	15.25
		DYT1 +ve	3	14.8
		DYT1 −ve	2	4.5
		DYT1 −ve	2	6.5
		Dystonia myoclonus syndrome	2	7.9
		DYT1 −ve	5	8.1
	Secondary dystonia	Term HIE	2	17.25
		Ex-Prem	5	5
		Kernicterus + deafness	4	5.75
		Kernicterus	5	2.75
		Term HIE	5	13.5
		Ex-Prem	4	11.6
		Term CP	4	12.5
		Methylmalonic acidaemia	5	5.2
		Ex-Prem	5	9.5
		Ex-Prem	5	9
		Glutaric aciduria	5	17.75
		Presumed neurometabolic	5	5.25
		Mitochondrial disorder	4	8.5
		Lesch Nyhan disease	5	12.9
		PKAN +ve	5	8.8
		PKAN +ve	5	7.0
		PKAN +ve	4	5.8
Intrathecal baclofen		Term HIE	5	17.5
		Ex-Prem	5	7.3
		Ex-Prem	5	4.75
		Term HIE	5	7.6
		Ex-Prem	5	12.25
		Ex-Prem	5	6.6
		Presumed neurometabolic	5	13.8
		Hypomyelination	4	6
		TBI	5	15.5
		Term HIE	5	12.76

*GFMCS* Gross Motor Function Classification System, *Ex-Prem* Ex-Prematurity (dystonia related to preterm birth), *CP* term CP, *HIE* hypoxic ichaemic encephalopathy, *PKAN* pantothenate kinase-associated neurodegeneration, *TBI* traumatic brain injury

### Imaging protocol

DWI sequences were obtained as part of the routine clinical work up of CAYP at our centre under evaluation for possible DBS or ITB surgery. All images were acquired on a 1.5T MRI scanner (Achieva, Philips, Best, The Netherlands) using an eight-channel phased array head coil. Diffusion-weighted single shot EPI sequences (FOV 190 × 190 mm, acquisition matrix 96 × 96, giving voxel size 1.98 × 1.98 mm, reconstructed to 112 × 112, giving a reconstructed voxel size of 1.76 mm × 1.76 mm, sense factor 2, 65 × 2 mm slices, slice gap 0 mm, half Fourier factor 0.62, 32 diffusion encoding directions with *B* values 0 and 1000s/mm^2^, TR 9592 ms, TE 89 ms, total scan time 05 min 54 s). In all cases, MRI was performed under general anaesthesia as the severity of movement disorder precluded acquisition in the awake state.

Voxel-wise statistical analysis of the fractional anisotropy (FA) data was carried out using Tract-Based Spatial Statistics (TBSS [15] part of FSL (http://www.fmrib.ox.ac.uk/fsl). TBSS projects all subjects’ FA data onto a mean FA tract skeleton, before applying voxel-wise cross-subject statistics. Firstly, diffusion-weighted images were registered to a non-diffusion weighted reference volume for correction of head motion and eddy currents. A mask was created to remove non-brain matter using the FMRIB Brain Extraction Tool (BET) [16]. FA images were created by fitting a tensor model to the raw diffusion data using FSL’s FDT tool, which uses a least-squares approach. FSL’s TBSS tool was used to align all subjects FA data into a common space, using the FMIRB nonlinear registration tool (FNIRT). Following registration, a mean FA image was created, using an FA threshold of 0.2, which was then thinned to create a mean FA skeleton, which represents the centre of all FA tracts common to the group. A value of 0.2 has most commonly been used in studies of adult cases and was chosen despite the young age of some subjects within the analysis to restrict the FA skeleton to white matter regions, as judged by visual inspection of the skeleton. Each subject’s aligned FA data was then projected onto this skeleton and the resulting data fed into voxel-wise cross subject statistics [15].

Three group analyses were performed:

1. CAYP selected for DBS surgery with primary dystonia (*n* = 14) were compared to those selected for ITB surgery (*n* = 10).
2. CAYP with a diagnosis of secondary dystonia selected for DBS surgery (*n* = 17) were compared to patients selected for ITB surgery (*n* = 10).
3. CAYP with a diagnosis of primary dystonia selected for DBS surgery (*n* = 14) were compared to those with a diagnosis of secondary dystonia selected for DBS surgery (*n* = 17).

The effect of group for each comparison was tested with age at scan included as a non-explanatory co-regressor, with threshold-free cluster enhancement with significance set at *p* < 0.05, fully corrected for multiple comparisons across space.

To illustrate the difference in FA values across the groups, FA values were extracted from the voxel with the most statistically significant difference between the groups. Given the focus on the CST with respect to choosing between DBS and ITB surgery, an estimate of tract-wide FA values for right and left CSTs was obtained from the average of all voxels in the mean FA skeleton which overlapped with the CST within the John Hopkins University (JHU) white matter tractography atlas [17]. To examine variation in FA along the length of this estimated CST, FA values were extracted from voxels within the CST at each separate slice level along the *Z*-axis.

Ethical approval was waived by Westminster Ethics Committee. Informed consent for all investigations was obtained from CAYP and/or their legal next of kin.

## Results

### Comparison between DBS and ITB groups

**Fig. 1:**
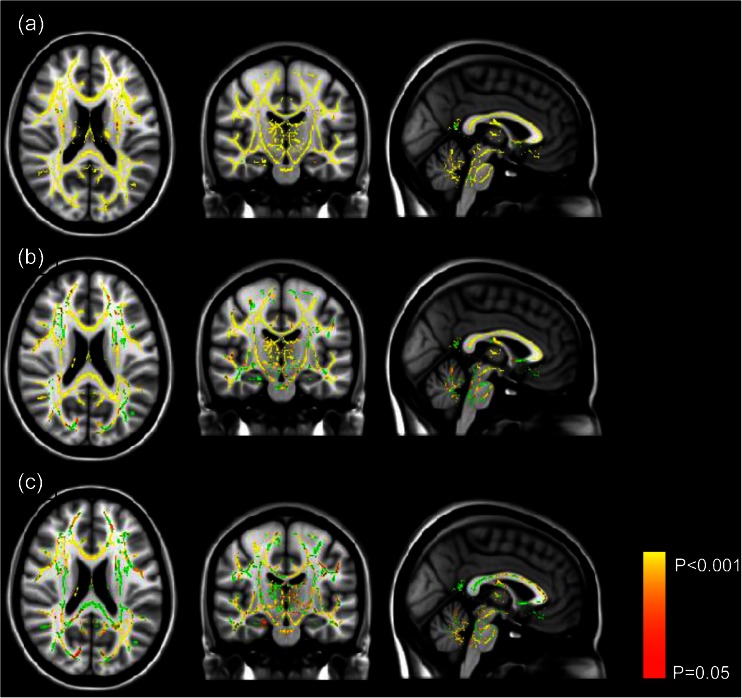
Results of Tract-Based Spatial Statistics (TBSS) analysis. Group comparisons in fractional anisotropy (FA) values are shown with age treated as non-explanatory co-regressors. The mean FA skeleton (shown in green) is overlaid on the MNI152 template. Regions of the skeleton in red-yellow represent significant differences (*P* < 0.05, family-wise error corrected). Differences shown are **a** areas of lower FA values in the ITB compared to primary dystonia DBS group, **b** areas of lower FA in the ITB compared to secondary dystonia DBS group and **c** areas of lower FA in the secondary dystonia compared to primary dystonia DBS groups

Results of the group comparison TBSS are shown in Fig. 1. Wide-spread (almost global) FA reduction was found in the ITB group compared to the primary dystonia DBS cohort (Fig. 1a). Less extensive, though still widespread, areas of reduced FA were seen comparing the ITB to the secondary dystonia DBS group (Fig. 1b), and the secondary dystonia DBS group compared to primary dystonia DBS group (Fig. 1c). These regions were not restricted to motor pathways.

**Fig. 2:**
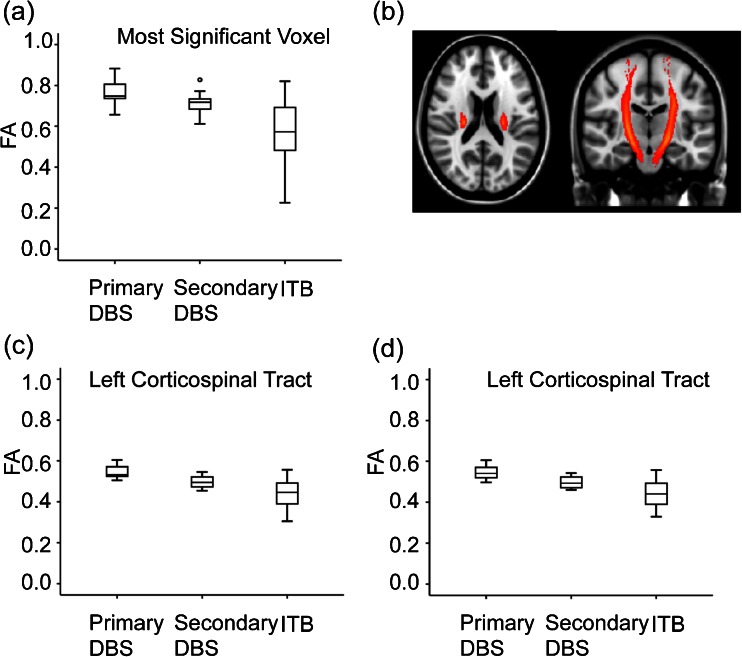
Comparison of FA values between surgical groups. **a** Box and Whisker plots of FA values extracted from the voxel with the most significant difference between ITB and secondary dystonia group. **b** John Hopkins University (JHU) white matter tractography atlas of the right and left corticospinal tract (CST), overlaid on the MNI152 template. **c**, **d** Estimate of mean tract FA values from left and right corticospinal tracts, obtained from intersection of voxels with the mean FA skeleton and the JHU tractography atlas CST

**Fig. 3:**
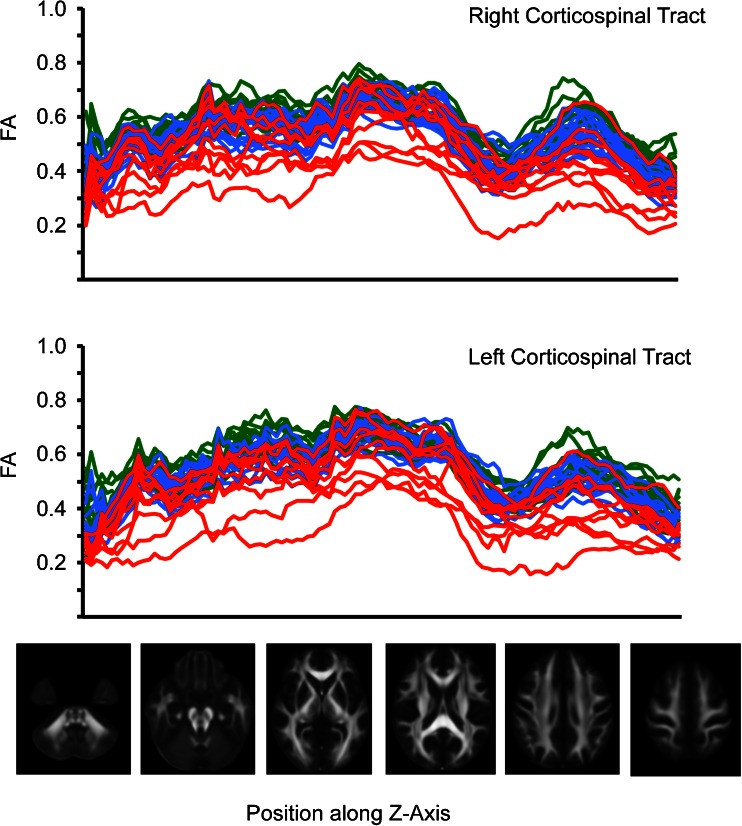
Fractional anisotropy (FA) values at different levels along the *Z*-axis of the CST for children with primary dystonia (*green*) and secondary dystonia (*blue*) undergoing DBS, and the ITB group (*red*). FA values are shown on the *y*-axis, with position along the CST on the *x*-axis, running left-right inferior-superior. Illustrative axial slices from the FMRIB58 FA template are displayed, indicating the anatomical position along the CST. Along the length of the tract highest values were seen in the primary group, and lowest in the ITB group, though there was considerable overlap between the groups, consistent with the box and whisker plots shown in Fig. 2

FA values extracted from voxels within the left and right CST are shown in Fig. 2. Considerable overlap was seen between the groups, and FA values in this region alone did not distinguish between groups. This was also the case for the voxel with the highest statistical significance in the analysis. The lowest FA values seen in the combined DBS group were 0.46 in the right CST and 0.48 in the left CST. This compared to median scores in the ITB group of 0.44 and 0.45 respectively. FA values along the Z-axis of the CST are shown in Fig. 3. A trend towards lower FA values globally along the length of the tract was seen in the ITB group, with highest values along the tract tending to be seen in the primary DBS group. As can been seen in this plot, FA at all levels along the tract demonstrated considerable overlap amongst groups.

## Discussion

TBSS demonstrated extensive differences in white matter microstructure between children and young people selected for ITB and those selected for DBS surgeries. Differences in FA values were found in all major white matter pathways. Lower regions of FA were found in candidates for ITB surgery compared to candidates for DBS surgery (both as a combined group and when separated into either primary or secondary dystonia groups). Comparing the cohorts of candidates for DBS surgery, significant differences were seen across the major white matter pathways. Lower FA values were seen in the secondary compared to primary dystonia group. ROI analysis from the PLIC would suggest that the magnitude of this difference was smaller.

Dystonia and spasticity arise from distinct pathophysiological processes. Previously considered a disease of the basal ganglia, increasing evidence suggests that dystonia may arise from perturbations at many points of the distributed motor network [18]. Pathophysiological processes found in primary dystonia include increased plasticity, decreased surround inhibition and increased cortical excitability [19]. It is unclear to what extent these mechanisms are shared by the secondary dystonias, though available evidence would suggest that pathophysiological differences exist [20]. Spasticity is a component of the upper motor neuron syndrome, arising due to hyperexcitable tonic stretch reflexes. This is, at least in part, due to loss of supraspinal control [21]. Insults to the brain resulting in areas of grey and white matter damage, e.g. following hypoxic ischaemic encephalopathy, may consequently give rise to a mixed hypertonicity. In practice, dichotomising CAYP with motor dysfunction arising from acquired brain injury into mutually exclusive categories of “spastic” or “dystonic” is overly reductive, as for many CAYP, both components of hypertonicity may be present to varying extents.

Neuroimaging correlates of motor phenotype in childhood have been most extensively studied in children with CP. Systematic reviews of neuroimaging findings in this population have demonstrated an imperfect relationship between motor phenotype and conventional imaging findings [22]. In children with hypertonicity arising from HIE, damage to the subthalamic nucleus strongly predicts a dyskinetic phenotype, whilst white matter damage near the paracentral lobule more strongly predicts a spastic phenotype [23].

White matter abnormalities have been more variably reported outside of the CST, with the superior and posterior thalamic radiations and corpus callosum most frequently examined [11]. Most studies have utilized ROI or tractography-based analysis, with only a small number of studies using whole-brain voxel-wise or TBSS analysis. In a study of 43 children with periventricular leukomalacia (PVL), areas of reduced FA were shown throughout most of the major white matter pathways in comparison to controls in a TBSS analysis [24]. Almost globally reduced FA values have been demonstrated in the neonatal period comparing neonates following hypoxic ischaemic delivery to healthy term controls [25]. Given the severe motor impairment in our ITB group (three with dystonia arising from HIE and four from preterm birth), it is unsurprising that they would exhibit such extensive areas of reduced FA, particularly in comparison to the primary dystonia group (with only minimal, variably reported abnormalities in FA described in this group compared to normal controls) [26].

Our findings are in contrast to the previous observation of more severe white matter disruption in dyskinetic compared to spastic CP [12, 13], instead suggesting that white matter disruption is more severe in spasticity. Possible explanations for the differences in findings may be differences in the severity of motor impairment compared in the previous atlas-based study (no GMFCS levels were provided in the ROI-based study) and the small sample sizes (only 10 CAYP in the ITB group in our study, and 7 CAYP with dyskinesia and 11 with spasticity in the previous atlas-based study). Furthermore, it is possible that differences in severity of underlying brain injury exist between different populations of CAYP scoring GMFCS 5, due to a “floor” effect of the grading system. We were unable to explore more fully any potential relationship between FA values and GMFCS levels in this cohort, due to the limited range of values seen in each dystonia aetiology group. An almost dichotomous split of GMFCS values was seen, with low GMFCS levels in the primary dystonia group, and uniformly high GMFCS levels in the secondary DBS and ITB groups.

One interpretation of our findings is that greater white matter integrity is required to express dystonia. This would be consistent with the observation that white matter injury is most commonly seen in CAYP with spastic CP, and also our previously reported findings of normal CMCT in the majority of children with dystonia and abnormal neuroimaging [9]. Abnormal CMCT has been reported as a feature of children with spasticity but not in those with extrapyramidal disorders [27]. We have previously demonstrated no relationship between DTI-metrics and CMCT in a large cohort of children with dystonia, including a number of cases included in this present study, suggesting that these measurements provide complementary information in the clinical assessment of children with motor disorders [28].

Whilst group-wise differences in FA between ITB and DBS groups were found, an overlap in value within the CST could be seen (Fig. 2), suggesting that tract-based or ROI FA values alone could not determine which intervention a child should be offered. Interestingly, FA values <0.4 for the CST were seen only in the ITB and not DBS group. A cut-off CST FA value of <0.5 has been suggested to predict more severe motor impairment in PVL, though this is derived from a tract-based metric [29]. In a comparison of 11 children with spastic quadriplegic CP and 12 children with spastic diplegic CP, low FA values of the CST to upper and lower limbs were demonstrated in the quadriplegic group (mean tract values 0.358 versus 0.430 to the upper limbs and 0.37 versus 0.434 to the lower limb) [30]. Yoshida et al. report a mean FA value in a left PLIC ROI of 0.584 in athetotic CP compared to 0.620 in the spastic CP group [12]. This compares to a value of 0.390 by Murakami et al. in a group of 10 subjects with severe PVL [29]. Caution must be taken in comparing DTI metrics between different studies due to differences in MRI acquisition, post-acquisition processing and potential large variations in manual placements of ROI.

Reduced white matter integrity was shown in the secondary dystonia compared to primary dystonia group. We hypothesise that this reduced white matter connectivity may account for differences to response to DBS seen comparing primary and secondary dystonia. Differences in FA values were seen very broadly across the major white matter tracts, reflective of the global injury to the brain arising from an insult such as HIE.

A number of limitations to our study must be acknowledged. DWI images were obtained during the routine clinical assessment of CAYP referred to our service. Sequence length was consequently constrained, restricting the number of directions to 32. Numbers of CAYP within each sub-group were relatively small, with a range of aetiologies in each group. This included a range of focal radiological abnormalities consistent with diagnosis shown in Table 1, e.g. hypoxic ischaemic encephalopathy, glutaric aciduria, etc. The range of aetiologies is consistent with the clinical heterogeneity of dystonia in childhood [31] and is also representative of the patient population under evaluation for movement disorder surgery. The CAYP in the secondary dystonia DBS and ITB groups represent a cohort with severe motor impairment, and it is unclear to what extent findings from this group may be generalized to children with milder impairment. One further important limitation is the lack of clinical outcome data at present to determine the benefit to patients clinically selected across the cohort into either “DBS” or “ITB” surgical groups. Further follow-up work is required to determine whether optimal patient selection was achieved, including an exploration of the relationship of FA values to “good” and “poor” responders in each surgical group.

## Conclusions

In a cohort of children and young people with hypertonicity of mixed origins undergoing evaluation for neurosurgical intervention, extensive areas of statistically significant lower FA were found comparing candidates for ITB and candidates for DBS surgery. DTI measures may therefore contribute to decision making for patient selection for movement disorder surgery. Significant differences were found in CAYP with secondary dystonia selected for DBS surgery compared to CAYP selected for ITB pump implants, suggesting that more extensive white matter injury is a feature of the spastic motor phenotype. Altered white matter microstructure was found across almost all of the major white matter pathways of the brain, which could possibly explain the reduced responsiveness to interventions such as DBS in this group.
